# Examining teachers’ attitudes towards inclusive education for all: development of a new scale

**DOI:** 10.1186/s40359-026-04478-6

**Published:** 2026-04-06

**Authors:** Stephan Kielblock, Stuart Woodcock, John Ehrich

**Affiliations:** 1 Carl von Ossietzky Universität Oldenburg, Oldenburg, Germany; 2 Edith Cowan University, Perth, Australia; 3 Macquarie University, Sydney, Australia

**Keywords:** Inclusive education, Attitudes, Teachers, Measurement, Scale

## Abstract

Teachers’ attitudes towards inclusive education are widely recognised as a key indicator of the inclusiveness of classroom environments. However, existing instruments inadequately capture teachers’ mindsets in relation to inclusive education for *all* learners. This study addresses this gap by developing and validating a new cross-cultural measurement instrument: the Attitudes Towards Inclusion for All scale (ATIFA), in both English (ATIFA-EN) and German (ATIFA-DE). Data were collected from pre-service and in-service teachers in Australia (*n* = 146) and Germany (*n* = 238). Exploratory factor analysis and Rasch modelling identified four robust subscales—Philosophy, Practice, Social Inclusion, and Support—with strong psychometric properties. Evidence of reliability and construct validity was further supported through correlations with teacher self-efficacy. The ATIFA provides a valid and reliable tool for assessing teachers’ attitudes towards inclusive education for all and offers a foundation for future cross-cultural research and policy development.

**Supplementary Information:**

The online version contains supplementary material available at 10.1186/s40359-026-04478-6.

## Introduction

Since its foundation in 1945, the United Nations Educational, Scientific and Cultural Organization (UNESCO) strives for inclusive education for all. In the 1990s, the developments toward inclusive education for all children gained momentum. UNESCO emphasised that “regular schools […] are the most effective means of combating discriminatory attitudes, creating welcoming communities, building an inclusive society and achieving education for all” [87], p. ix). Until today, inclusive education for all stands for quality education for all children, “irrespective of sex, age, race, colour, ethnicity, language, religion, political or other opinion, national or social origin, property or birth, as well as persons with disabilities, migrants, indigenous peoples, and children and youth” [88], p. 25).

### Definition of inclusive education

Starting in the 1990s, researchers adopted this view of inclusion being concerned with “the education of *all* children and young people” [15], p. 1, emphasis added). Inclusive education *for all* means “the right of *all* children to access, presence, participation and success in their local regular school” [78], p. 8, emphasis added). In this perspective, inclusive education is a shift from deficit, diagnosis, and labels to quality education for all children, framed in terms of diversity [3, 19, 68, 76, 84]. In this view, each student is valued as an individual with certain strengths and potential. Valuing learner diversity enriches teaching and classroom experiences for all students.

### The role of teachers and their attitudes

Research has underlined the importance of teachers for positive development of their students [36, 40, 59, 72]. The teachers’ mindsets and attitudes are emphasised as an important driver for more inclusive teaching strategies [49, 80]. Accordingly, policies claim that teachers have to understand the concept of inclusive education in order to cater for all children [19, 88].

According to theory, behaviour is a function of behavioural intents, attitudes, subjective norms and the perceived behavioural control [4, 5]. Amongst these concepts, particularly attitudes have been in the focus of research [6, 89]. Attitudes are a tendency of an individual to evaluate an object in a favourable or unfavourable way [24]. In other words, if a teacher has an unfavourable view on inclusion for all, it may be unlikely that the necessary action and effort to inclusive teaching will be displayed by the teacher. In this way, the teachers’ attitudes towards inclusive education for all can be considered a proxy for the inclusiveness of the learning environment that the teacher creates for his or her students. Accordingly, it is important to obtain empirical evidence regarding the inclusive mindsets of teachers.

## Key approaches to measuring teachers’ attitudes towards inclusive education

A large variety of studies have utilised different instruments to measure teachers’ attitudes towards inclusive education [28, 44]. In a recent review, Kielblock and Woodcock [44] analysed which attitude instruments are used in current empirical studies on teachers’ attitudes towards inclusive education. They found five instruments most commonly used: the Attitudes Towards Inclusive Education Scale (ATIES [94, 96],), the Sentiments, Attitudes, and Concerns about Inclusive Education scale (SACIE [56],), its revised form (SACIE-R [32],), the Opinions Relative to Mainstreaming scales (ORM [51],) and the Opinions Relative to Integration scale (ORI [8],).

A closer look into the content and the structure of these scales reveals two main strands of measuring the teachers’ attitudes towards inclusive education. First, there is a multi-dimensional approach to attitudes towards inclusive education, focusing on different aspects of inclusion, such as the philosophy of inclusion, inclusive practices, or the placement of students with special educational needs and/or disabilities (SEND) in the regular classroom. Second, there might be doubts, if a teacher would respond to the same items in the same way, if s/he had different types of SEND in mind, when completing the survey instrument. Hence, a second strand of measuring teachers’ attitudes towards inclusive education responds to the demand to differentiate different kinds of SEND, when asking if the placement of students with SEND in the regular classroom might be appropriate.

### Multi-dimensional approach

#### ORM Opinions Relative to Mainstreaming (1979)

In the 1970s, Larrivee and Cook [51] developed an instrument that was attempted to capture the attitudes of teachers towards “mainstreaming mildly handicapped children” (p. 315). At that time, mainstreaming was debated in the United States and the question was pressing, what general education teachers were thinking about students with SEND in the regular classroom, and what factors might lead to positive attitudes. The analysis was carried out with a sample of 941 regular-classroom teachers (K–12) in the United States. The final version had 30 items and five dimensions: (1) General philosophy (e.g., Item 30 “The presence of special-needs students will promote acceptance of differences on the part of regular students.”), (2) Classroom behaviour of ‘special needs children’ (e.g., Item 14 “Most special-needs children are well behaved in the classroom.”), (3) Perceived ability to teach ‘the special needs child’ (e.g. Item 16 “Regular-classroom teachers have sufficient training to teach children with special needs.”), (4) Classroom management with special needs children (e.g. Item 17 “Special-needs students will monopolize the teacher’s time.”), and (5) Academic and social growth of the special needs child (e.g., Item 2 “The needs of handicapped students can best be served through special, separate classes.”). This scale is known as the ‘Opinions Relative to Mainstreaming’ (ORM) scale and it was used in various subsequent studies. Even more recently, under the umbrella term of ‘inclusive education’, this scale was utilised in many studies (such as [45, 48, 50, 65]).

#### ORI Opinions Relative to Integration (1995)

A widely utilised further development of the ORM was published by Antonak and Larrivee [8]. During the 1980s, the idea of ‘mainstreaming’ was more and more replaced by the ‘integration’ discourse. Hence, the measurement instrument needed to capture new terminologies that became more and more relevant. ‘Mainstreaming’ became ‘integration’, ‘special-needs students’ was turned into ‘students with disabilities’ and ‘regular’ or ‘normal’ were changed into ‘general’. A sample of 433 pre-service teachers from the United States completed the new survey instrument and four factors were found. (1) Benefits of integration (e.g., Item 17 “The integration of students with disabilities can be beneficial for students without disabilities.”), (2) Integrated classroom management (e.g., Item 18 “Students with disabilities are likely to create confusion in the general classroom.”), (3) Perceived ability to teach students with disabilities (e.g., Item 19 “General-classroom teachers have sufficient training to teach students with disabilities.”), (4) Special versus integrated general education (e.g., Item 5 “Students with disabilities can be best served in general classrooms.”). This instrument is commonly referred to as the ‘Opinions Relative to Integration’ (ORI) scale. In the last 25 years, the ORI was used widely for measuring teachers’ attitudes (such as [61, 71, 77, 90]).

A comparison between the original ORM and the ORI scale reveals that the ‘general philosophy’ (ORM) and the ‘benefits of integration’ (ORI) are relatively similar. In addition, the ‘perceived ability to teach the special needs child/students with disabilities’ is present in both variations of the scale (ORM, ORI). The dimension ‘classroom behaviour of special needs children’ (ORM) and ‘classroom management with special needs children’ (ORM) became just one dimension in the ORI (‘integrated classroom management’).

#### TAIS Teachers’ Attitudes towards Inclusion Scale (2015)

The ORI was not the only update of the ORM. As mentioned, in the 1990s ‘inclusive education’ started to gain momentum as a new way of thinking. As ‘inclusion’ started to displace ‘integration’ more and more, a further update of the wording was needed. Monsen et al. [62] presented a psychometrically sound update, which they referred to as ‘Teachers’ Attitudes towards Inclusion Scale’ (TAIS). Data were collected from teachers in England (*n* = 95). Through factor analysis, Monsen et al. [62] found four factors: (1) problems of inclusion of SEN children in mainstream classes (e.g., Item 7 “It is difficult to maintain order in a normal classroom that contains an SEN child.”), (2) social benefits for all of the inclusion of SEN pupils in mainstream classes (e.g., Item 10 “Isolation in a special class has a negative effect on the social and emotional development of an SEN child.”), (3) implications of inclusion for teaching practice (e.g., Item 27 “Inclusion of SEN children necessitates extensive retraining of regular classroom teachers.”), (4) implications for teachers addressing the needs of children with SEN (e.g., Item 22 “SEN children need to be told exactly what to do and how to do it.”).

In relation to the original ORM, the TAIS – generally – represents the classroom behaviour of students with SEND (ORM dimension 2, TAIS dimension 1), teachers’ training in teaching students with SEND (ORM dimension 3, TAIS dimension 3), teachers’ classroom management with students with SEND (ORM dimension 4, TAIS dimension 4) and the benefits for students with SEND being taught in the regular classroom (ORM dimension 5, TAIS dimension 2). Only the ‘general philosophy’ (ORM dimension 1) was not a factor in the TAIS.

#### Summary

The ‘multi-dimensional’ strand of measuring teachers’ attitudes originated in the United States context in the 1970s. The focus of the instruments (and many other existing instruments that are similar) is to allow insights into teachers’ thinking regarding the philosophy, practices, training, and benefits of teaching students with and without SEND together in the general classroom.

### Approach that differentiates between types of SEND

#### ATMS Attitudes towards Mainstreaming Scale (1980)

At the same time when the ORM was developed, another research group in the United States developed a different approach to measuring teachers’ attitudes. As mentioned above, in the 1980s the “movement to integrate disabled students into the regular classroom (mainstreaming)” [13], p. 199) gained momentum in the United States. Berryman et al. [13] attempted to find statements that captured “general disabilities categories” (p. 200). These statements should be not too complicated so that “subjects other than educators of exceptional children” (p. 200) can answer them. Hence, they developed 22 items, which included the label of the disability and a description of the behaviour that might result from the disability, and they asked if students with such type of SEND should be taught in the regular classroom. An example of such an item is: “Blind students, who cannot read standard printed material” (p. 200) should be in the regular classroom. The sample comprised 161 higher education students (to be teachers, school counsellors, or school administrators) in the United States. Factor analysis demonstrated four factors: (1) learning capability (e.g., Item 9 “Physically handicapped students confined to wheelchairs should be in regular classrooms.”), (2) general mainstreaming (e.g., Item 1 “In general, mainstreaming is a desirable educational practice.”), (3) severe disability (e.g., Item 6 “Blind students who cannot read standard printed material should be in the regular classroom”), (4) social behaviour (e.g., Item 16 “Students with behaviour disorders who cannot readily control their own behaviour should be in regular classrooms.”).

In a re-analysis, Berryman & Neal [12] found three factors: (1) Learning Capability, (2) General Mainstreaming, which were similar to the previous analysis, and (3) Traditional Limiting Disability, which was termed ‘Classroom Functioning’ in later studies [10, 11, 37].

#### ATIES Attitudes towards Inclusive Education Scale (1991)

In 1991, Wilczenski [93, 95] wanted to describe the ‘attitudes towards mainstreaming’ held by undergraduate education students. A sample of 229 education majors was drawn in a college in the United States. The individuals completed the 18-item ATMS [10, 11]. Through factor analysis, Wilczenski [93] found three dimensions, which were – generally – in line with the ATMS dimensions: (1) Learning Capability (e.g. “Students with diabetes should be in regular classrooms”), (2) General Mainstreaming and Limiting Disabilities (e.g. “Students should have the right to be in regular classrooms.” or “Educable mentally retarded students should be in regular classrooms.”), and (3) Specific Disabilities (e.g. “Students who present persistent discipline problems should be in regular classrooms.”).

Wilczenski [94] attempted to develop the ATMS(-R) further and focus more on the “impact of integrating students with disabilities in the regular class program” (p. 307). Hence, Wilczenski [94] drew a sample of regular class teachers (*n* = 301) and undergraduate elementary pre-service teachers (*n* = 144) in the United States and asked them to complete 32 items, which “dealt with the feasibility of a regular class placement for students requiring physical, academic, behavioural, or social accommodations” (p. 307). The analysis found sufficient psychometric properties for 16 items on four factors: (1) Physical accommodations, (2) Academic modifications of the regular class curriculum, (3) Disruptive behaviour, and (4) Social participation. However, a re-analysis of the factor structure, using a new sample of 445 regular class teachers from the United States, clearly demonstrated the unidimensionality of the scale [96]. This unidimensional instrument is commonly referred to as the ‘Attitudes towards Inclusive Education Scale’ (ATIES). It had a large influence on research on teachers’ attitudes towards inclusive education. Hence, it has been used in many studies (such as [14, 58, 60, 83]).

The comparison of the ATIES and the original ATMS reveals two differences: First, the ATMS-dimension, which captured the general philosophy of mainstreaming (ATMS ‘general mainstreaming’), was not present in the ATIES anymore. The instrument was completely focused on the placement for students identified with particular types of SEND. Second, the ATIES turned out to be genuinely unidimensional.

#### SACIE(-R) Sentiments, Attitudes and Concerns about Inclusive Education (2007)

Loreman et al. [56] combined the ATIES with other dimensions of the teachers’ thinking, namely, the sentiments, attitudes, and the concerns regarding inclusive education. In order to capture these three dimensions, Loreman et al. [56] developed an instrument that combined the Interactions with People with Disabilities Scale (IDP [34, 35],), the Concerns about Inclusive Education Scale (CIES [74],) and the ATIES [94, 96]. Pre-service teachers across Australia (*n* = 57), Canada (*n* = 191), Singapore (*n* = 102) and Hong Kong (*n* = 438) completed this combination of instruments. Factor analysis and different steps of refinement led to seven attitude statements, which were thought to measure the attitudes towards inclusive education (e.g., Item 5 “Students who have difficulty expressing their thoughts verbally should be in regular classes.”).

The whole instrument, including the sentiments and the concerns dimension, is commonly referred to as the ‘Sentiments, Attitudes and Concerns about Inclusive Education’ (SACIE) scale. The SACIE was utilised by many studies, (such as [18, 25, 66, 73]). This instrument is one of the few instruments in this field, which was genuinely constructed for cross-cultural use.

Using a sample of 542 pre-service teachers across Hong Kong, Canada, India and the United States, Forlin et al. [32] developed a revised version of this scale, the SACIE-R. The SACIE-R scale had three dimensions: sentiments, concerns and attitudes. The attitude dimension comprised five items.

Regarding the content of the attitude-part of the SACIE-R, the sub-scale asks, if it is feasible to have a student in the regular class, who (1) has difficulty expressing her or his thoughts verbally, (2) is inattentive, (3) requires communicative technologies (e.g., Braille/sign language), (4) who frequently fails exams, and (5) who needs an individualised academic programme. The SACIE-R is widely utilised for measuring the teachers’ attitudes, concerns, and sentiments in different studies (such as [47, 64, 70, 100]).

#### Summary

The strand to differentiate the types of SEND has a long tradition in the measurement of teachers’ attitudes towards inclusive education; it goes back at least to the 1970 s in the United States, where it was supposed to support the implementation of mainstreaming. The focus of the instruments is to ask the teachers which types of SEND seem to allow a child to be part of the regular classroom.

### Challenges regarding the measurement of teachers’ attitudes towards inclusive education – the research problem

Two strands of measuring the teachers’ attitudes towards inclusive education have been described so far: The multi-dimensional approach allowed insights into teachers’ thinking regarding the philosophy, practices, training, and benefits of teaching students with and without SEND together in the general classroom. On the other hand, the approach that differentiated between types of SEND allowed insights into how teachers might respond differently regarding the question of placement of a child with SEND in the regular classroom, depending on the type of SEND.

The brief history of the measurement of teachers’ attitudes towards (mainstreaming, then integration and now) inclusive education has two important results. On the one hand, the instruments discussed, and many other instruments that have been developed within these two strands, gained important insights into teachers’ attitudes. These sound scales were thoroughly developed and refined over many important iterations. On the other hand, the brief history of the two strands point to the fact that the scales were developed basically in regard to updates of the terminology. The underlying philosophy of these scales—namely, the placement of students with SEND in general classrooms—has remained largely unchanged. Consequently, current measurement approaches originated, and are still deeply rooted in the mainstreaming discourse in the United States starting in the late 1970s.

At the beginning of this paper, inclusive education was introduced as quality education *for all* children [3, 15, 78, 88]. It seemed that in the 1990 s, global policy [86, 87] and research [2, 15] started to embrace the diverse strengths and capabilities of all learners and the pressing question was, how to best cater for all diverse learners in the classroom. Such a view on inclusive education *for all* needs to be represented in teachers’ attitude instruments. A recent comprehensive review of instruments that measured the teachers’ attitudes towards inclusive education revealed that there is not a single instrument available that fully captures inclusive education *for all* [44]. The vast majority of available studies on teachers’ attitudes towards inclusive education seem to assume a clear cut between regular and special education, and between regular and special students.

This is a large challenge for research and policy. Research in the United States in the 1970 s responded timely to the new demands of the ‘P.L. 94–142’ law from 1975. At the end of the 1970 s, sound research and new instruments in this particular area were available. Yet, research on inclusive education *for all* still does not seem to be available. There seems to be an urgent need to develop a new psychometrically sound instrument to measure the teachers’ attitudes toward inclusive education *for all*. This instrument should be valid in different countries, with different cultural backgrounds, and with different languages. Hence, the research question of this study is, how teachers’ attitudes towards inclusive education *for all* can be measured.

## Methods

A new measurement instrument for cross-cultural use should be developed for and in multinational, multicultural and multilingual contexts [39]. Hence, Australia and Germany were selected for the present study, as two countries, which were generally comparable with each other, but which differ on these three dimensions (multinational, multicultural and multilingual). While Australia had already undergone major steps towards inclusive education for all [19] and its school system was built on neo-liberal values [29, 43], Germany had in large parts separated special schools for students who were identified with SEND [9, 42] and was mostly considered as social-democratic in terms of schooling [85].

### Participants

Considering different recommendations regarding an adequate sample size [23, 30, 46], and with the goal in mind to develop a new instrument, about 100–200 completed questionnaires for each country was thought to be obtained. The final sample comprised 384 pre- and in-service teachers from Australia (*n* = 146) and Germany (*n* = 238).

Most teachers in the sample were female (Australia 70.3%, Germany 80.1%), which was in line with the official statistics in Australia [1] and Germany [81]. A larger proportion of teachers in the sample were up to 30 years old (Australia 65.5%, Germany 72.9), and 61 and 71 percent were pre-service teachers in Australia and Germany, respectively. Especially in Australia, more teachers in the sample were teaching or were in training to teach in the primary sector (69.6%), while in Germany, this proportion was slightly lower (57.7%).

### Instruments

#### Attitudes

In order to develop a new instrument to measure teachers’ attitudes towards inclusive education for all, an extensive search for previous relevant questionnaires was carried out. In 14 papers, 46 relevant items were found that could be used as relevant indicators for the new instrument. These items represented a considerable range of topics: Inclusion and equality is a right; inclusive education is desirable (e.g., leads to valuable experiences, permits academic and social progression, leads to social inclusion); adaptations and adjustments are needed (e.g., with regard to the assessment); supports are necessary for carrying out more inclusive practices (e.g., additional personnel). This variety of topics, as it was represented by the items, seemed to cover a relevant range of aspects of inclusive education for all.

The wording of these items was analysed in cycles of critical item revisions. In addition, written feedback regarding the readability and clarity of the items was gathered from two pre-service teachers and two in-service teachers from Australia. Some items were merged, as these were too similar and some items were discarded. These iterations narrowed down the number of items to 38.

For translating and adapting survey instruments from one language into another, commonly back-translation [17, 22, 91] and split-forward translation [38, 69, 97] are recommended. In our study, we utilised both approaches: The 38-item instrument was translated from English to German by a professional translator and by the present researchers separately (split-forward) and a professional translator translated the professional German version back into English (back-translation). Intensive comparisons and discussions led to one reconciled German version.

In order to critically assess the readability and clarity, two pre-service teachers and two in-service teachers commented on the questionnaire in written form. In order to examine how teachers interact with the different items and what they think the items actually mean, one pre-service and one in-service teacher took part in think-aloud interviews [20, 26, 27, 53, 98]. The participants of these interviews were encouraged to verbalize their thoughts when completing the questionnaire, and these verbalisations were analysed as being indicators of the ongoing cognitive processes while working through the items. The wording of the German items was slightly adjusted due to the results of these pre-tests.

According to the decision framework for selecting a response scale format [92] a 7-point scale was utilised as a response format. This framework recommends 5- or 7-point scales for scale development studies, as they provide sufficient response differentiation while maintaining reliability. In addition, the odd number of response options allows for a neutral midpoint, which has been shown to reduce misresponses to reversed items compared to scales without a middle category. The end-points of the scale were labelled with ‘very strongly disagree’ and ‘very strongly agree’. In addition, all seven answer options had a number attached, supporting the interpretation of unfavourableness with negative numbers (− 3, − 2, and − 1), positive numbers for favourableness (+ 1, + 2, and + 3), and zero for the neutral neither disagree nor agree (as recommended e.g. by [54]). In the data, the seven answer options were coded as 1 = very strongly disagree to 7 = very strongly agree.

#### Self-efficacy

In accordance with former studies (such as [16, 33, 41, 57, 63, 80, 99]) and in accordance with the theory [4, 5, 31], the hypothesis was formulated that the teachers’ self-efficacy in carrying out inclusive practices was positively related to the teachers’ attitudes towards inclusive education for all. In order to validate the new attitude instrument, the Teacher Efficacy for Inclusive Practices (TEIP [75], scale was utilised in the present study. The TEIP was suitable, because this instrument measured the teachers’ ability to include all learners and studies in English and German language reported a sufficient reliability [41, 75]. However, in five of the eighteen TEIP-items “students with disabilities” was changed into “all students”.

The TEIP measures teachers’ self-efficacy on three dimensions: Efficacy in Managing Behaviour (e.g. “I am confident in my ability to prevent disruptive behaviour in the classroom before it occurs.”), Efficacy in Collaboration (e.g. “I am able to work jointly with other professionals and staff (e.g., aides, other teachers) to teach all students in the classroom”), and Efficacy to use Inclusive Instructions (e.g. “I can use a variety of assessment strategies (e.g., portfolio assessment, modified tests, performance-based assessment, etc.).”). Multiple-group confirmatory factor analysis demonstrated insufficient fit regarding the proposed factor structure (*χ*^*2*^ (264, *n* = 382) = 800.88, CFI = 0.82, TLI = 0.80, RMSEA = 0.10, SRMR = 0.08). Hence, another multiple-group confirmatory factor analysis was calculated using only the three best-fitting items for each of the three dimensions. This model was acceptable (*χ*^*2*^ (48, *n* = 378) = 106.45, CFI = 0.96, TLI = 0.94, RMSEA = 0.08, SRMR = 0.05).

The internal consistency was sufficient for the ‘managing behaviour’ dimension (Cronbach’s alpha: Australia 0.84, Germany 0.83), for the ‘collaboration’ dimension (Cronbach’s alpha: Australia 0.79, Germany 0.73), and for the ‘using inclusive instruction’ dimension (Cronbach’s alpha: Australia 0.75), yet, for the latter dimension, the internal consistency was relatively low (Cronbach’s alpha: Germany 0.59).

#### Demographics

For the examination of differential item functioning (DIF), country (Germany vs Australia), gender (male vs female), and program (primary vs secondary) were utilised.

### Procedures

The present study examined the attitudes of teachers across all different stages of their professional careers (initial training, newly qualified teacher/probationary year, in-service, further training, etc.). The data collection procedures were similar in Australia and Germany: Pre-service teachers were approached through the unit (course) convenors of selected core units (courses), and, if approved, all students within the selected units (courses) were invited to partake in the study. The in-service teachers were approached through the school principals. If a principal consented, all teachers within the school were asked for participation in the study. Ethics and all necessary applications to conduct the study in the different schools and universities were approved before data collection.

### Data analyses

Sample size was adequate for all statistical tests **(***n* = 384). The sample size was adequate for factor analysis (i.e., > 200 considered good; [21]). The sample size for Rasch analysis with rating scale parameterization was adequate (i.e., *n* = 200 is sufficient for stable item calibrations within 0.5 logits at 99% confidence; [55]).

The analysis of the data was carried out in three steps: First, Kaiser–Meyer–Olkin (KMO) index and Bartlett’s test of sphericity were used to examine, if the data were sufficient for factor analysis. Principal axis factoring (PAF) with Varimax orthogonal rotation was utilised to examine the factor structure. Parallel analysis (PA) was used to indicate the number of factors to extract.

Second, Rasch analysis was used to test the linear measurement properties of the subscales (i.e., factor structures) attained from the prior EFA. The Rasch model is a probabilistic mathematical model which is commonly used to affirm the linear measurement properties of ordinal (Likert scale) data. For a full description of the Rasch model and its application and statistical interpretation see Tennant and Conaghan [82].

A series of Rasch analyses using the polytomous Rasch model (PRM) were run on the subscales. Rasch Unidimensional Measurement Modelling (RUMM) 2030 software [7] was used for all analyses. Person parameter estimates were obtained by the Weighted Maximum Likelihood method. Extreme scores (defined as 0 or all correct) were removed from all analyses. Log-likelihood ratio tests indicated partial credit parametrisation be applied per Rasch analysis (all *p*s < 0.02). Well-functioning of items to the PRM, individually and or collectively indicate 1) ordered thresholds, 2) model fit, 3) acceptable reliability indices, 4) individual item fit, 5) item invariance tested via differential item functioning (DIF), 6) response dependency, and 7) unidimensionality (evidence that only a single latent trait is being measured).

Third, inter-correlations of the attitude and self-efficacy subscales were calculated in order to validate the obtained attitude subscales.

## Results

### Factor analysis

In order to determine the internal structure of the 38-item inclusion instrument two common factor analyses (i.e., PAF, principal axis factoring) were conducted. A preliminary PAF indicated an orthogonal rotation method (i.e., based on examination of the communal factor matrix (all factor correlations < 0.5)). Also, a parallel analysis (PA) using the Monte Carlo simulation approach [52] indicated 5 factors to retain in the EFA. A final PAF using Varimax orthogonal rotation indicated acceptable sampling with all values for individual variables above > 0.5, and an acceptable Kaiser–Meyer–Olkin (KMO) index (0.921). Bartlett’s test of sphericity indicated that item correlations were sufficient for EFA, *Χ*^2^(703) = 6381, *p* < 0.001 [30]. The PA derived five-factor solution explained 52.6% of the variance. The scree plot also indicated a five-factor solution. The five factor solution included Factor 1 *Philosophy* with 14 items (6, 13, 14, 15, 16, 17, 18, 19, 21, 25, 30, 32, 36, 38); Factor 2 *Practice* with seven items (2, 8, 12, 23, 24, 27, 31); Factor 3 *Social Inclusion* with six items (1, 10, 20, 28, 35, 37); and Factor 4 *Support* with five items (5, 7, 9, 22, 33). Factor 5 consisted of only two items (3 and 11), which did not indicate a clear, conceptually coherent dimension. Item 3 refers to teachers’ feelings of being overwhelmed when differentiating instruction, whereas Item 11 relates to beliefs about the necessity of labelling students to provide appropriate education. Given the limited number of items and the lack of a coherent underlying construct, this factor was not considered further in the subsequent Rasch analyses. All items loaded well (standardised factor loadings > 0.4; see Table 1).

**Table 1:** Factor loadings of the 38-item inclusion scale

	Factor				
	1	2	3	4	5
6. All students will receive appropriate education and related services in inclusive education	.427				
*13. All children should be educated in the inclusive classroom	.657				
*14. All children are capable of learning in inclusive settings	.685				
*15. Inclusion represents a negative change in our education system	.596				
*16. I believe that with the right supports in place inclusion can work	.623				
17. I feel that inclusive education is a practical idea in my country	.518				
18. Separating students is not necessary to provide a quality education to them	.432				
*19. I believe that any student can learn in an inclusive school if the curriculum is adapted to meet their individual needs	.686				
21. The philosophy of inclusion cannot be implemented in ‘real world’ practices	.531				
*25. I feel differentiated adjustments can be carried out in an inclusive classroom	.496				
*30. Inclusion is the best way to meet the needs of all students	.594				
*32. Diversity within the classroom enriches the learning environment	.535				
36. It is a valuable experience for all children to be educated in inclusive classrooms	.697				
*38. Inclusive education ultimately leads to social inclusion	.611				
*2. Effective Teachers are able to meet the needs of all children in the classes they teach		.524			
*8. It is possible to organise classes in a way that is suitable for all children		.427			
*12. The differentiated practices that inclusive education would require cannot be achieved		.491			
23. I get frustrated when I have to adapt the curriculum to meet the individual needs of all students		.422			
*24. I am willing to adapt the curriculum to meet the individual needs of all students within inclusive classrooms		.518			
*27. It is too difficult to accommodate all students’ differences in an inclusive classroom		.569			
31. Good teachers can differentiate their practices so that they can teach all students in their class/es		.518			
*1. Inclusion facilitates socially appropriate behaviour for all students			.532		
*10. Inclusion will foster acceptance of differences among students			.613		
20. I feel that external support services are a waste of time			.531		
28. I am willing to adapt the assessment of individual students in order for inclusive education to take place			.488		
*35. Inclusion will foster understanding of differences among students			.597		
37. Working collaboratively with parents plays a major part in the success of inclusion			.467		
*5. I feel there are adequate personnel from outside school to support me to address the unique educational needs of all students				.447	
7. I feel from my experience that there is support for inclusion from the Education Department/Board				.672	
*9. I feel there are adequate personnel within school to support me to address the unique educational needs of all students				.543	
22. I feel from my experience that the Education Department/Board supports efforts at including all students into the classroom				.680	
*33. I feel there are adequate resources to support me to address the unique educational needs of all students				.491	
3. I get overwhelmed when I have to differentiate to cater for all of the students ‘ needs in my classroom					.502
11. Labelling students (e.g. gender, race, ethnicity, disability, language, socio-economic status) is necessary to provide a quality education to them					.411

Factor loadings <.40 have been supressed

^*^Items marked with an asterisk were retained in the final instrument following the Rasch analyses

### Rasch analysis

#### Philosophy subscale

A Rasch analysis was run on the Philosophy subscale for the items (6, 13, 14, 15, 16, 17, 18, 19, 21, 25, 30, 32, 36, & 38). The results indicated disordered thresholds in items (13, 14, 17, 30, 32, & 36). Disordered thresholds indicate incongruent choices of response categories relative to the ability of respondents, that is higher ability persons choosing lower than expected level response categories and vice versa. These disordered thresholds were resolved by collapsing the response categories (1 + 2) for items 13, 14, 30, and 36 and collapsing the response categories (1 + 2 + 3) for items 17 and 32.

Model fit, as indicated by a non-significant (*p* > 0.05) item trait interaction (*Χ*^2^ statistic), indicated misfit to the model, *Χ*^*2*^(70) = 210.6, *p* < 0.001. This indicates an inconsistent hierarchical ordering of items across the latent trait [82]. The person separation index (PSI) which is a reliability index interpreted similarly to a Cronbach alpha (i.e., > 0.7 is acceptable), indicated a reliable scale (0.91).

Individual fit of items to the model is determined by attainment of acceptable fit residuals (− 2.5 to + 2.5); chi-square and *F*-test statistics (ANOVAs) where an insignificant *p*-value (> 0.05) indicates item fit; and inspection of the item characteristic curves (ICCs). ICCs are graphs which plot observed data against the Rasch estimates depicted as a theoretical s-shaped curved. Proximity of the observed data to the theoretical curve indicates acceptable item fit. All items fit the model with the exception of items 6 (fit residual = 3.672), 17 (fit residual = 2.982), 18 (fit residual = 9.439, *Χ*^*2*^ = 76.284, *p* < 0.001; *F* = 9.613, *p* < 0.001), 21 (fit residual = 3.156, *Χ*^*2*^ = 21.412, *p* < 0.001; *F* = 3.374, *p* < 0.005) and 36 (fit residual = − 3.213, *Χ*^*2*^ = 20.820, *p* < 0.001; *F* = 6.168, *p* < 0.001).

Item invariance was determined by DIF testing to determine response bias (i.e., when groups of persons with the same levels of the latent trait respond differently). DIF is tested by running two-way ANOVAs on the standardised residuals and applying Bonferroni adjustments. Consistent responses across class intervals indicate uniform DIF while inconsistent responses indicate non-uniform DIF. When significant uniform DIF is detected the item can be resolved by splitting, which involves creating two separate items, one per group. If the calibration of separate group-related items does not adversely impact on the scale, the item can be retained. In the case of non-significant DIF the item cannot be split and the usual procedure involves discarding the item [67]. DIF was run on three group factors – Country (Germany vs Australia), Gender (male vs female), and Program (primary vs secondary). After Bonferroni adjustment (alpha = 0.001), significant uniform DIF was found for Country in items 13 (*F* = 19.07, *p* < 0.001), 18 (*F* = 19.53, *p* < 0.001) and 30 (*F* = 22.73, *p* < 0.001); and Program in items 16 (*F* = 4.61, *p* < 0.001), 18 (*F* = 8.44, *p* < 0.001), and 36 (*F* = 5.75, *p* < 0.001). Significant non-uniform DIF was found for Country in items 16 (*F* = 4.72, *p* < 0.001), 18 (*F* = 9.66, *p* < 0.001), and 36 (*F* = 6.14, *p* < 0.001); and for Gender in items 16 (*F* = 4.50, *p* < 0.001), 18 (*F* = 9.41, *p* < 0.001), 36 (*F* = 5.99, *p* < 0.001).

Response dependency and unidimensionality were both tested by a principal components analysis (PCA) conducted on the standardised residuals. Response dependency is the effect of one item on the response of another or that items are too closely related to one another. An examination of the PCA residual correlation matrix indicated no items were closely related (all correlations < 0.3). Unidimensionality was tested by Smith’s [79] *t*-test approach which involves running multiple *t*-tests for each person measure on a set of positively and negatively loaded items attained from the first principal component loading. A large number of cases (> 5%) significantly different at the 5% level indicates evidence of multidimensionality. Significant differences were detected in 36 cases indicating 9.38% at the 5% level indicating multiple dimensions in the data.

Overall, the 14-item Philosophy subscale indicated poor psychometric functioning as indicated by misfit to the Rasch model, multiple misfitting items, items which were not invariant, and multiple dimensions in the data. However, on removal of misfitting items (6, 17, 18, 21, 36) a well-functioning subscale of 9-items (13, 14, 15, 16, 19, 25, 30, 32, & 38) was attained with acceptable model fit, *Χ*^*2*^(45) = 52.1, *p* = 0.22, and good reliability (PSI = 0.88) (see Table 2). Disordered thresholds were found in items 30 and 32 which were resolved by collapsing the response categories of (1 + 2) for item 30 and (1 + 2 + 3) for item 32. All 9 items fit the Rasch model (see Table 3). DIF analysis on the group factors of Country, Gender, and Program indicated no significant non-uniform however significant uniform DIF was found for Country in items 13 (*F* = 11.93, *p* < 0.001), 16 (*F* = 12.09, *p* < 0.001), and 30 (*F* = 13.30, *p* < 0.001), and for Program in item 19 (*F* = 12.99, *p* < 0.001). No significant uniform DIF was found for the group factor Gender. However, the items with significant uniform DIF were resolved by splitting. This did not adversely impact the scale (i.e., all PSIs > 0.88), hence these items were retained in the subscale.

**Table 2:** Rasch analysis summary statistics of the four subscales of the inclusion scale

	Item trait Interaction			
Subscale	Value (*df*)	*p*	PSI	Unidimensionality
Philosophy	52.1 (45)	.22	.88	7.55%**
Practice	30.1 (25)	.22	.75	3.66%
Social Inclusion	18.9 (15)	.23	.70	1.84%
Support	14.2 (15)	.51	.62	1.05%

*ps <.05 are statistically significant. These results were attained after the removal of misfitting items (items were removed from the Philosophy (i.e., items 6, 17, 18, 21, & 36), Social Inclusion (i.e., items 20 & 37), Practice (i.e., items 23 & 31), and Support subscales (i.e., items 7 & 22). The percentages in the unidimensionality column represent the proportion of cases which differ at the 5% level (> 5%** indicates multidimensionality)

**Table 3:** Fit of the individual items to the Rasch model of the Philosophy, Practice, Social Inclusion, and Support subscales

Item Number	Fit Residual	ChiSq	P	F	P
**Philosophy**					
6	**3.672**	17.048	.004	3.056	.010
*13	0.886	2.444	.785	0.414	.839
*14	0.050	3.530	.619	0.715	.612
*15	1.707	5.339	.376	1.124	.347
*16	− 1.324	9.137	.104	2.03	.074
17	**2.982**	6.085	.298	1.055	.385
18	**9.439**	76.284	.000*	9.613	.000*
*19	1.222	9.050	.107	2.012	.076
21	**3.156**	21.412	.000*	3.374	.005
*25	1.913	4.889	.430	1.023	.404
*30	− 1.543	7.218	.205	2.077	.068
*32	0.53	6.143	.293	1.233	.293
36	− **3.213**	20.82	.000*	6.168	.000*
*38	− 0.435	4.392	.494	0.845	.519
**Practice**					
*2	2.017	5.92	.314	1.24	.290
*8	− 0.495	9.455	.092	2.15	.059
*12	0.795	7.034	.218	1.932	.088
23	**3.621**	21.793	.000*	3.728	.002*
*24	0.629	4.198	.521	0.928	.463
*27	− 0.536	3.451	.631	1.315	.257
31	− 1.553	13.638	.018	3.544	.003*
**Social Inclusion**					
*1	1.332	3.561	.614	0.802	.549
*10	− 0.328	9.152	.103	2.93	.013
20	**3.270**	17.394	.003*	2.861	.015
28	**2.847**	7.855	.164	1.707	.132
*35	0.354	6.167	.290	1.893	.095
37	**3.241**	11.105	.049	2.110	.063
**Support**					
*5	0.603	10.500	.062	2.660	.022
7	0.392	2.910	.714	0.635	.672
*9	− 1.206	6.429	.266	1.747	.123
22	**2.890**	4.676	.456	0.701	.623
*33	0.764	3.731	.588	0.600	.699

Items with fit residuals < − 2.5 and > + 2.5 are considered misfitting and in bold print. Bonferroni adjusted *p*-values are significant at * *p* <.0036;.05/14 (Philosophy); * *p* <.0071;.05/7 (Practice); * *p* <.0083;.05/6 (Social Inclusion); * *p* <.01;.05/5 (Support)

^*^Items marked with an asterisk were retained in the final instrument following the Rasch analyses

A PCA on the standardised residuals indicated no response dependency (all correlations < 0.3). Unidimensionality tests indicated that 7.55% of cases were significantly different (i.e., > 5%) at the 5% level suggesting some multi-dimensionality. Overall, the 9-item Philosophy subscale functioned well according to the Rasch model.

#### Practice subscale

Rasch analysis on the Practice subscale for items (2, 8, 12, 23, 24, 27, & 31) indicated acceptable model fit, *Χ*^*2*^(25) = 30.1, *p* = 0.22, after the removal of two misfitting items, item 23 (fit residual = 3.621, *Χ*^*2*^ = 21.79, *p* < 0.001; *F* = 3.73, *p* < 0.003) and item 31 (*F* = 3.54, *p* < 0.004) (see Table 2). All thresholds were ordered with the exception of item 2 which was resolved through the collapsing of the (1 + 2) response categories. The PSI indicated reasonable reliability (0.75). All remaining items indicated good fit to the Rasch model (see Table 3). DIF analysis on the group factors of Country, Gender, and Program, indicated no significant non-uniform DIF however significant uniform DIF was found for Country in items 2 (*F* = 19.11, *p* < 0.001), 8 (*F* = 30.62, *p* < 0.001), and 27 (*F* = 14.31, *p* < 0.001). These items were resolved by splitting (all PSIs > 0.76 after splitting) and hence were retained in the subscale. No significant DIF was found for Gender and Program.

A PCA on standardised residuals indicated a unidimensional subscale, that is, less than 5% of differences in person locations were detected (3.66%) following Smith’s t-test method. No response dependency was found (all correlations < 0.3). Overall, the subscale indicated well-functioning according to the Rasch model.

#### Social inclusion subscale

**Fig. 1:**
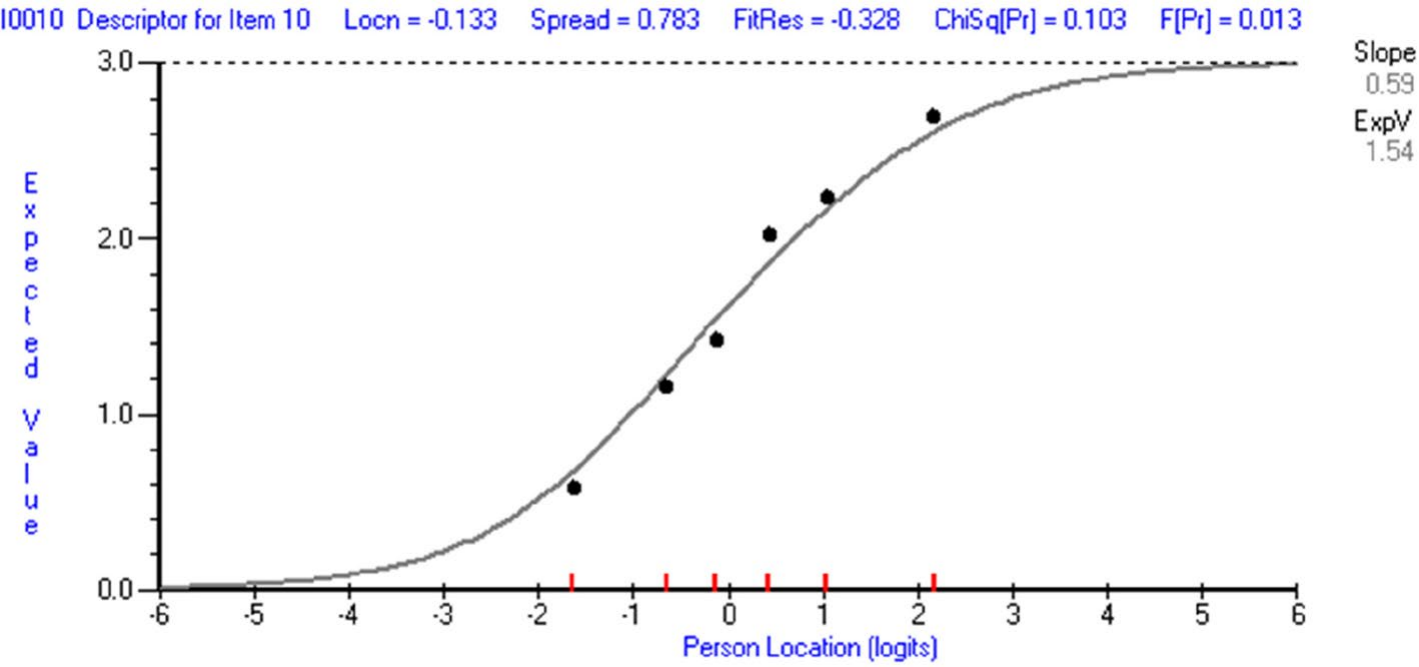
ICC of item 10 (Inclusion will foster acceptance of differences among students). Note. In this graph the observed data (depicted as black dots) closely follow the Rasch theoretical estimates (depicted as the sigmoid curve) indicating a well-functioning item

An initial Rasch analysis on the subscale Social Inclusion for items (1, 10, 20, 28, 35, & 37) indicated misfit to the model, *Χ*^*2*^(30) = 68.25, *p* < 0.001. However, a well-functioning 3-item subscale was found after the removal of misfitting items 20 (fit residual = 3.270, *Χ*^*2*^ = 17.394, *p* < 0.004), 28 (fit residual = 2.847) and item 37 (fit residual = 3.241). The data fit the model, *Χ*^*2*^(15) = 18.88, *p* = 0.23, and the scale indicated acceptable reliability (PSI = 0.70) (see Table 2). The three remaining items (item 1, 10, and 35) indicated disordered thresholds. Thresholds became ordered upon collapsing the response categories of (1 + 2 + 3) of each item. All items indicated good fit to the Rasch model except for item 10 which indicated some misfit with a significant *F* statistic (*F* = 2.93, *p* = 0.01) (see Table 3). However, inspection of the item’s ICC indicated a well-functioning item (see Fig. 1).

DIF analysis on the group factors of Country, Gender, and Program indicated neither significant uniform nor non-uniform DIF. A PCA on the standardised residuals indicated no response dependency (all correlations < 0.3). Unidimensionality tests indicated that 1.84% of cases were significantly different (i.e., > 5%) at the 5% level suggesting a unidimensional scale. Overall, the 3-item subscale indicated a well-functioning scale.

#### Support subscale

Rasch analysis on the Support subscale (items 5, 7, 9, 22, & 33) indicated acceptable model fit, *Χ*^*2*^(15) = 14.2, *p* = 0.51, after the removal of two misfitting and response dependent items (7 & 22) (see Table 2). While the reliability index was lower (PSI = 0.63) and reflects the reduced item count (following the removal of items 7 and 22), the subscale demonstrates sufficient reliability for exploratory group-level comparisons. Individual item misfit was found in Item 22 only (Fit residual = 2.890) (see Table 3). Disordered thresholds were found in item 33 which was resolved by collapsing the response categories (1 + 2 + 3). DIF analysis on the group factors of Country, Gender, and Program indicated neither significant uniform DIF nor non-uniform DIF. A PCA on the standardised residuals and examination of the residual correlation matrix indicated no response dependency except for items 7 and 22 (> 0.3). Undimensionality testing following Smith’s *t*-test method indicated a unidimensional scale with only 1.05% of person locations differing at the 5% level.

In summary, Rasch analysis indicated four well-functioning subscales of the multi-dimensional inclusion scale, a 9-item Philosophy (13, 14, 15, 16, 19, 25, 30, 32, & 38), a 5-item Practice (2, 8, 12, 24, & 27), a 3-item Social Inclusion (1, 10, & 35), and a 3-item Support (5, 9, 33) subscale.

### Reliability and validity analysis

#### Reliability

These four subscales were tested regarding their internal consistency using Cronbach’s alpha. All four subscales resulted in very acceptable Cronbach’s alpha (Philosophy, 0.90; Practice, 0.78; Social Inclusion, 0.80; and, Support, 0.70). The overall Cronbach’s alpha including all 20 items resulted in 0.91.

#### Intercorrelations

All subscales of the new measurement instrument are significantly correlated with each other (Table 4). Philosophy, Practice and Social Inclusion are highly correlated with each other (above 0.5). The Support dimension is less strongly related with the other three (0.32, 0.36 and 0.21 for Philosophy, Practice and Social Inclusion, respectively). The interrelatedness of the dimensions of the new instrument demonstrates the validity of the measurement. Regarding the self-efficacy instrument (TEIP), Table 4 shows that all three dimensions are correlated, which suggests that the dimensions coherently measure self-efficacy.

**Table 4:** Correlations of the four attitude dimensions with three self-efficacy dimensions

	1	2	3	4	5	6	7
1. Philosophy^a^	–-						
2. Practice^a^	.71**	–-					
3. Social Inclusion^a^	.73**	.51**	–-				
4. Support^a^	.32**	.36**	.21**	–-			
5. Managing Behaviour^b^	.08	.14**	−.01	.00	–-		
6. Collaboration^b^	.34**	.37**	.26**	.09	.61**	–-	
7. Inclusive Instruction^b^	.21**	.25**	.13*	−.01	.68**	.72**	–-

n_min._ = 371; * <.05. ** <.01; ^a^attitude dimensions; ^b^self-efficacy dimensions

The comparison between the attitude and the self-efficacy dimensions reveals that the three dimensions of the attitudes (namely, Philosophy, Practice and Social Inclusion) are significantly related to two self-efficacy dimensions (namely, using inclusive instruction and collaboration). Regarding these dimensions, the correlations indicate a valid measurement of the attitudes and self-efficacy.

## Discussion

### Interpretation of the results

This study developed a new, psychometrically sound measurement instrument, which is valid in different countries, with different cultural backgrounds, and with different languages. While other attitude instruments in the area of inclusive education focus students with SEND (like the ORM, the ORI or the TAIS) or differentiated different types of SEND (like the ATMS, the ATIES or the SACIE), the newly developed instrument (Attitudes Towards Inclusion For All – ATIFA scale; see Appendix) is multidimensional with a clear focus on all students. Its dimensions cover a variety of important facets of inclusive education for all from the perspective of teachers. Hence, the instrument is ready to be used for studies on teachers’ views on inclusive education for all.

Each step of the development was carried out with rigor: (1) Relevant items were found through an extensive literature search, a careful revision of potential items, and a thorough translation from English into German. (2) Before collecting data, face-validity and content adequacy was established through feedback from pre-service and in-service teachers from Australia and Germany. (3) Then, empirical data from two countries were collected and analysed. Through factor analysis and Rasch analysis, four subscales were found and examined in detail: Philosophy, Practice, Social Inclusion and Support. (4) The internal consistency was established for all subscales with a Cronbach’s alpha ranging from 0.70 to 0.90. (5) A comparison with three dimensions of an established instrument for measuring teachers’ self-efficacy towards inclusive education demonstrated validity. (6) A replication of the study is still pending. Yet, the sample for developing the instrument comprised different countries, cultural backgrounds, and languages. In addition, the data comprised teachers in different stages of their careers. This is the foundation for the usability of the instrument and the stability of the factor structure.

DIF analyses indicated some items functioned differently across countries or across pre-service versus in-service teachers. Uniform DIF could be statistically accounted for through item splitting. Nevertheless, the findings suggest that some aspects of the attitudes to inclusive education for all may partly be shaped by contextual and cultural factors, which future cross-national studies should continue to examine.

### Limitations

Although a considerable amount of effort was invested in making informed decisions, limitations need to be acknowledged. A limitation is that the present study was focused on two larger cities in Australia and Germany. Although these contexts were found to be adequate for the development of the new instrument, the samples of teachers did not incorporate the broader population. More specifically, the samples were drawn from metropolitan teacher education and school contexts, which may differ from rural or regional settings in terms of institutional structures and experiences with inclusive education. In addition, young and pre-service teachers were overrepresented in the sample, which may limit the generalisability of the findings to more experienced teachers.

Furthermore, five items of the TEIP instrument were slightly adapted by replacing “students with disabilities” with “all students”. While conceptually aligned with the broader understanding of inclusive education in this study, this modification may have influenced the psychometric properties of the scale, namely, the relatively low reliability of the “using inclusive instruction” dimension in Germany. Finally, all analyses were conducted on the same dataset. Future studies should therefore examine the stability of the scale structure using independent samples.

### Implications

The findings of the present study seem to be promising for strengthening the perspective on inclusive education for all. This is important for teachers and their teaching practices. Across the globe, many teachers were trained to focus on the average student, but inclusive education for all draws attention to responding to issues as they pertain to the presence, participation and achievement of all of the students. In this way, the concept of inclusive education for all, which was defined and consistently used in the present study, might help teachers (and others, e.g., principals, parents, policy makers) to adopt the vision of inclusive education for all and adapt their practices accordingly, to be able to cater for all students.

An advantage of the quantitative approach, used in this study is that it enables the collection of data in schools about the inclusive thoughts of the staff. A school for example would gain valuable insights into how positive teachers think about the different facets as they pertain to inclusive education for all. According to this evidence, specific school developments could be initiated, and the new instrument could even be used to monitor if these interventions have the desired effects. In this sense, the new instrument could be used in schools to promote interventions that facilitate the development of inclusive thinking and practices. For example, a school could administer the scale to its teaching staff prior to implementing a professional development program on inclusive pedagogy. The results could help identify specific areas in which teachers feel more or less confident or hold stronger or weaker attitudes (e.g., regarding participation or achievement of all students). Professional development activities could then be targeted to these areas, and the scale could be administered again after the training to examine whether teachers’ attitudes toward inclusive education for all have changed.

In addition, for the Education Departments/Boards and for the institutions that provide pre-service teacher training (such as universities) the results of the present study suggest consideration to changes and re-developments of policies and programs so that they are in line with inclusive education for all. The instrument that the present study developed might also be used to collect information from teachers about the impact these changes are making. In accordance, further changes could be initiated, and the effects could again be monitored by using the instrument, which was developed in the present study.

There are also implications for further research. A great amount of studies on teachers’ attitudes towards inclusive education use instruments which focus on students with SEND or on different types of SEND. This reminds the teachers repeatedly that there would be a need to think about particular students differently compared to all of the other students that are considered to be normal. Yet, in a way, the content of a questionnaire must be considered an intervention: Through the completion of questionnaires on inclusive education the teachers’ attention is drawn on issues as they pertain to some students particularly, and not all students. The findings of the present study indicate that there is now a way to measure the teachers’ attitudes toward inclusive education for all. Future research should utilise the instrument developed in the present study in different cultural and educational contexts and with diverse teacher populations in order to further examine its applicability and generalisability.

## Conclusion

This study contributes to the field of inclusive education by developing a psychometrically sound, cross-cultural instrument that captures teachers’ attitudes towards inclusive education for all learners. Unlike existing measures, the ATIFA shifts the focus from specific student groups to a comprehensive view of inclusion, reflecting contemporary educational priorities. The identified four-factor structure demonstrates strong reliability and validity across two national contexts. The instrument offers practical value for research, teacher education, and school development by enabling the systematic assessment and monitoring of inclusive attitudes. Future research should replicate these findings in diverse contexts and further examine the scale’s longitudinal stability and applicability.

## Supplementary Information

Supplementary Material 1. Appendix.

## Data Availability

The datasets generated and analysed during the current study are not publicly available due to ethical and legal restrictions, as they contain sensitive information from human participants and were collected under informed consent agreements that did not permit public data sharing. In accordance with institutional ethics approvals and data protection regulations, the data can be made available upon reasonable request to the corresponding author.
