# Supplementary material for: Examining teachers’ attitudes towards inclusive education for all: development of a new scale
Source: BMC Psychol. 2026 Apr 6;14:503. doi: 10.1186/s40359-026-04478-6 (PMC13067759; doi:10.1186/s40359-026-04478-6)
Supplement: Supplementary file 1 — Supplementary Material 1. Appendix. [file 40359_2026_4478_MOESM1_ESM.docx]

# Appendix

| **Attitudes Towards Inclusive Education for All (ATIFA-EN)** | \|  \|  \| \| --- \| --- \| \| *-3*  *-2*  *-1*  *0*  *+1*  *+2*  *+3* \| *= very strongly disagree*  *= strongly disagree*  *= disagree*  *= neither disagree nor agree*  *= agree*  *= strongly agree*  *= very strongly agree* \| \|  \|  \| |
| --- | --- | --- | --- | --- | --- | --- | --- |
| *These statements ask to what extent you think inclusive settings are best for all students. Please indicate below (by crossing one box in each row) how strongly you disagree or agree with the different statements.* |  |

|  |  | very strongly **disagree** | | | |  |  | |  | very strongly **agree** | | | |
| --- | --- | --- | --- | --- | --- | --- | --- | --- | --- | --- | --- | --- | --- |
|  |  |  | |  | | | |  | | | |  | |
|  |  |  | **-**3 | | **-**2 | **-**1 | 0 | | **+**1 | **+**2 | **+**3 | |  |
|  |  |  |  | |  |  |  | |  |  |  | |  |
| Inclusion facilitates socially appropriate behaviour for all students. | | | ☐ | | ☐ | ☐ | ☐ | | ☐ | ☐ | ☐ | |  |
|  |  |  |  | |  |  |  | |  |  |  | |  |
| Effective Teachers are able to meet the needs of all children in the classes they teach. | | | ☐ | | ☐ | ☐ | ☐ | | ☐ | ☐ | ☐ | |  |
|  |  |  |  | |  |  |  | |  |  |  | |  |
| I feel there are adequate personnel from outside school to support me to address the unique educational needs of all students. | | | ☐ | | ☐ | ☐ | ☐ | | ☐ | ☐ | ☐ | |  |
|  |  |  |  | |  |  |  | |  |  |  | |  |
| All children are capable of learning in inclusive settings. | | | ☐ | | ☐ | ☐ | ☐ | | ☐ | ☐ | ☐ | |  |
|  |  |  |  | |  |  |  | |  |  |  | |  |
| It is possible to organise classes in a way that is suitable for all children. | | | ☐ | | ☐ | ☐ | ☐ | | ☐ | ☐ | ☐ | |  |
|  |  |  |  | |  |  |  | |  |  |  | |  |
| I believe that with the right supports in place inclusion can work. | | | ☐ | | ☐ | ☐ | ☐ | | ☐ | ☐ | ☐ | |  |
|  |  |  |  | |  |  |  | |  |  |  | |  |
| I am willing to adapt the curriculum to meet the individual needs of all students within inclusive classrooms. | | | ☐ | | ☐ | ☐ | ☐ | | ☐ | ☐ | ☐ | |  |
|  |  |  |  | |  |  |  | |  |  |  | |  |
| Inclusion will foster understanding of differences among students. | | | ☐ | | ☐ | ☐ | ☐ | | ☐ | ☐ | ☐ | |  |
|  |  |  |  | |  |  |  | |  |  |  | |  |
| Inclusion is the best way to meet the needs of all students. | | | ☐ | | ☐ | ☐ | ☐ | | ☐ | ☐ | ☐ | |  |
|  |  |  |  | |  |  |  | |  |  |  | |  |
| I feel there are adequate resources to support me to address the unique educational needs of all students. | | | ☐ | | ☐ | ☐ | ☐ | | ☐ | ☐ | ☐ | |  |
|  |  |  |  | |  |  |  | |  |  |  | |  |
| It is too difficult to accommodate all students’ differences in an inclusive classroom. | | | ☐ | | ☐ | ☐ | ☐ | | ☐ | ☐ | ☐ | |  |
|  |  |  |  | |  |  |  | |  |  |  | |  |
| All children should be educated in the inclusive classroom. | | | ☐ | | ☐ | ☐ | ☐ | | ☐ | ☐ | ☐ | |  |
|  |  |  |  | |  |  |  | |  |  |  | |  |
| Diversity within the classroom enriches the learning environment. | | | ☐ | | ☐ | ☐ | ☐ | | ☐ | ☐ | ☐ | |  |
|  |  |  |  | |  |  |  | |  |  |  | |  |
| I feel there are adequate personnel within school to support me to address the unique educational needs of all students. | | | ☐ | | ☐ | ☐ | ☐ | | ☐ | ☐ | ☐ | |  |
|  |  |  |  | |  |  |  | |  |  |  | |  |
| I believe that any student can learn in an inclusive school if the curriculum is adapted to meet their individual needs. | | | ☐ | | ☐ | ☐ | ☐ | | ☐ | ☐ | ☐ | |  |
|  |  |  |  | |  |  |  | |  |  |  | |  |
| The differentiated practices that inclusive education would require cannot be achieved. | | | ☐ | | ☐ | ☐ | ☐ | | ☐ | ☐ | ☐ | |  |
|  |  |  |  | |  |  |  | |  |  |  | |  |
| Inclusion represents a negative change in our education system. | | | ☐ | | ☐ | ☐ | ☐ | | ☐ | ☐ | ☐ | |  |
|  |  |  |  | |  |  |  | |  |  |  | |  |
| Inclusion will foster acceptance of differences among students. | | | ☐ | | ☐ | ☐ | ☐ | | ☐ | ☐ | ☐ | |  |
|  |  |  |  | |  |  |  | |  |  |  | |  |
| I feel differentiated adjustments can be carried out in an inclusive classroom. | | | ☐ | | ☐ | ☐ | ☐ | | ☐ | ☐ | ☐ | |  |
|  |  |  |  | |  |  |  | |  |  |  | |  |
| Inclusive education ultimately leads to social inclusion. | | | ☐ | | ☐ | ☐ | ☐ | | ☐ | ☐ | ☐ | |  |
|  |  |  |  | |  |  |  | |  |  |  | |  |

| **Einstellung zu inklusiver Bildung für alle (ATIFA-DE)** | \|  \|  \| \| --- \| --- \| \| *-3*  *-2*  *-1*  *0*  *+1*  *+2*  *+3* \| *= lehne sehr stark ab*  *= lehne stark ab*  *= lehne ab*  *= weder noch*  *= stimme zu*  *= stimme stark zu*  *= stimme sehr stark zu* \| \|  \|  \| |
| --- | --- | --- | --- | --- | --- | --- | --- |
| *Die Aussagen im Folgenden fragen, inwieweit Ihrer Meinung nach inklusive Settings für alle Schüler:innen das Beste sind. Bitte kreuzen Sie in jeder Reihe an, in welchem Maße Sie den unterschiedlichen Aussagen zustimmen oder diese ablehnen.* |  |

|  |  | **lehne** sehr stark **ab** | | | |  |  | |  | **stimme** sehr stark **zu** | | | |
| --- | --- | --- | --- | --- | --- | --- | --- | --- | --- | --- | --- | --- | --- |
|  |  |  | |  | | | |  | | | |  | |
|  |  |  | **-**3 | | **-**2 | **-**1 | 0 | | **+**1 | **+**2 | **+**3 | |  |
|  |  |  |  | |  |  |  | |  |  |  | |  |
| Inklusion fördert das sozial angemessene Verhalten aller Schüler:innen. | | | ☐ | | ☐ | ☐ | ☐ | | ☐ | ☐ | ☐ | |  |
|  |  |  |  | |  |  |  | |  |  |  | |  |
| Gute Lehrer:innen können die Bedürfnisse aller Schüler:innen ihrer Klasse erfüllen. | | | ☐ | | ☐ | ☐ | ☐ | | ☐ | ☐ | ☐ | |  |
|  |  |  |  | |  |  |  | |  |  |  | |  |
| Meinem Gefühl nach gibt es geeignetes externes Personal, das mir hilft, auf die einzigartigen Bildungsbedürfnisse aller Schüler:innen einzugehen. | | | ☐ | | ☐ | ☐ | ☐ | | ☐ | ☐ | ☐ | |  |
|  |  |  |  | |  |  |  | |  |  |  | |  |
| Alle Kinder können in einem inklusiven Umfeld lernen. | | | ☐ | | ☐ | ☐ | ☐ | | ☐ | ☐ | ☐ | |  |
|  |  |  |  | |  |  |  | |  |  |  | |  |
| Es ist möglich, Klassen so zu organisieren, dass der Unterricht für alle Kinder geeignet ist. | | | ☐ | | ☐ | ☐ | ☐ | | ☐ | ☐ | ☐ | |  |
|  |  |  |  | |  |  |  | |  |  |  | |  |
| Ich glaube, dass Inklusion funktionieren kann, wenn die richtigen Unterstützungsleistungen vorhanden sind. | | | ☐ | | ☐ | ☐ | ☐ | | ☐ | ☐ | ☐ | |  |
|  |  |  |  | |  |  |  | |  |  |  | |  |
| Ich bin bereit, in inklusiven Klassen das Curriculum auf die individuellen Bedürfnisse aller Schüler:innen hin anzupassen. | | | ☐ | | ☐ | ☐ | ☐ | | ☐ | ☐ | ☐ | |  |
|  |  |  |  | |  |  |  | |  |  |  | |  |
| Inklusion fördert das Verständnis für die Unterschiede zwischen Schüler:innen. | | | ☐ | | ☐ | ☐ | ☐ | | ☐ | ☐ | ☐ | |  |
|  |  |  |  | |  |  |  | |  |  |  | |  |
| Inklusion ist der beste Weg, um die Bedürfnisse aller Schüler:innen zu erfüllen. | | | ☐ | | ☐ | ☐ | ☐ | | ☐ | ☐ | ☐ | |  |
|  |  |  |  | |  |  |  | |  |  |  | |  |
| Meiner Meinung nach gibt es geeignete Ressourcen, die mir helfen, auf die individuellen Lernbedürfnisse aller Schüler:innen einzugehen. | | | ☐ | | ☐ | ☐ | ☐ | | ☐ | ☐ | ☐ | |  |
|  |  |  |  | |  |  |  | |  |  |  | |  |
| Alle Unterschiede der Schüler:innen einer inklusiven Klasse zu berücksichtigen, ist zu schwierig. | | | ☐ | | ☐ | ☐ | ☐ | | ☐ | ☐ | ☐ | |  |
|  |  |  |  | |  |  |  | |  |  |  | |  |
| Alle Kinder sollten inklusiv unterrichtet werden. | | | ☐ | | ☐ | ☐ | ☐ | | ☐ | ☐ | ☐ | |  |
|  |  |  |  | |  |  |  | |  |  |  | |  |
| Vielfalt in der Klasse bereichert die Lernumgebung. | | | ☐ | | ☐ | ☐ | ☐ | | ☐ | ☐ | ☐ | |  |
|  |  |  |  | |  |  |  | |  |  |  | |  |
| Ich denke, es gibt geeignetes internes Personal, das mir hilft, auf die einzigartigen Bildungsbedürfnisse aller Schüler:innen einzugehen. | | | ☐ | | ☐ | ☐ | ☐ | | ☐ | ☐ | ☐ | |  |
|  |  |  |  | |  |  |  | |  |  |  | |  |
| Ich glaube, dass jede/r Schüler/in an einer inklusiven Schule lernen kann, wenn das Curriculum auf die individuellen Bedürfnisse abgestimmt wird. | | | ☐ | | ☐ | ☐ | ☐ | | ☐ | ☐ | ☐ | |  |
|  |  |  |  | |  |  |  | |  |  |  | |  |
| Ein differenzierter Unterricht, wie er für inklusive Bildung nötig ist, kann nicht geleistet werden. | | | ☐ | | ☐ | ☐ | ☐ | | ☐ | ☐ | ☐ | |  |
|  |  |  |  | |  |  |  | |  |  |  | |  |
| Inklusion ist eine negative Veränderung in unserem Bildungssystem. | | | ☐ | | ☐ | ☐ | ☐ | | ☐ | ☐ | ☐ | |  |
|  |  |  |  | |  |  |  | |  |  |  | |  |
| Inklusion fördert die Akzeptanz von Unterschieden zwischen den Schüler:innen. | | | ☐ | | ☐ | ☐ | ☐ | | ☐ | ☐ | ☐ | |  |
|  |  |  |  | |  |  |  | |  |  |  | |  |
| Ich bin der Meinung, dass Differenzierung in einer inklusiven Klasse möglich ist. | | | ☐ | | ☐ | ☐ | ☐ | | ☐ | ☐ | ☐ | |  |
|  |  |  |  | |  |  |  | |  |  |  | |  |
| Inklusive Bildung führt letztendlich zu sozialer Inklusion. | | | ☐ | | ☐ | ☐ | ☐ | | ☐ | ☐ | ☐ | |  |
|  |  |  |  | |  |  |  | |  |  |  | |  |
